# Evaluation of Heat-Induced Damage in Concrete Using Machine Learning of Ultrasonic Pulse Waves

**DOI:** 10.3390/ma15227914

**Published:** 2022-11-09

**Authors:** Ma. Doreen Esplana Candelaria, Nhoja Marie Miranda Chua, Seong-Hoon Kee

**Affiliations:** 1 Department of ICT Integrated Ocean Smart Cities Engineering, Dong-A University, Busan 49315, Korea; 2 Institute of Civil Engineering, University of the Philippines Diliman, Quezon City 1101, Philippines

**Keywords:** thermal damage, concrete, ultrasonic pulse waves, machine learning

## Abstract

This study investigated the applicability of using ultrasonic wave signals in detecting early fire damage in concrete. This study analyzed the reliability of using the linear (wave velocity) and nonlinear (coherence) parameters from ultrasonic pulse measurements and the applicability of machine learning in assessing the thermal damage of concrete cylinders. While machine learning has been used in some damage detections for concrete, its feasibility has not been fully investigated in classifying thermal damage. Data was collected from laboratory experiments using concrete specimens with three different water-to-binder ratios (0.54, 0.46, and 0.35). The specimens were subjected to different target temperatures (100 °C, 200 °C, 300 °C, 400 °C, and 600 °C) and another set of cylinders was subjected to room temperature (20 °C) to represent the normal temperature condition. It was observed that P-wave velocities increased by 0.1% to 10.44% when the concretes were heated to 100 °C, and then decreased continuously until 600 °C by 48.46% to 65.80%. Conversely, coherence showed a significant decrease after exposure to 100 °C but had fluctuating values in the range of 0.110 to 0.223 thereafter. In terms of classifying the thermal damage of concrete, machine learning yielded an accuracy of 76.0% while the use of P-wave velocity and coherence yielded accuracies of 30.26% and 32.31%, respectively.

## 1. Introduction

Thermal damages in concrete structures have a substantial influence in terms of social and economic impact. Between 2017 and 2019, about 368,500 residential building fires were reported in the United States, resulting in property losses of USD 8.1 billion [1]. In Japan, an average of 22,776 building fires were recorded, with an average of USD 578,354 loss in damaged property in the past decade [2] while at least 5000 fire cases are reported in the EU every day, costing up to EUR 126 billion each year [3]. In South Korea, an estimated average of 42,000 residential building fires and an average of USD 730 million in property loss have been recorded in the past decade [4]. If feasible, doing repairs is the most cost-effective and time-efficient option, and the most appropriate restoration procedures are determined after an initial inspection of the fire-exposed concrete [5]. From the standpoint of having safe and reliable operations of these structures, it is important to understand the variation in the effects of fire damage on the different properties of concrete. It is critical to create a reliable method for assessing the condition of heat-damaged concrete in structures and, if necessary, determining proper operation and maintenance strategies.

Heat-induced concrete damage may be evaluated in a variety of ways. The preliminary level of assessment is based mostly on optical inspection and sounding (hammer tapping). However, optical inspections give only surface information, and the outcomes are dependent on the inspector’s experience [6]. Similarly, the sounding process is subjective in nature, and reliable findings require skilled inspectors [7]. In the laboratory, a variety of analytical procedures might be utilized (e.g., colorimetry, X-ray diffraction analysis, differential thermal analysis, chemical analysis, dilatometry, porosimeter, or micro-crack density analysis) [8]. However, they need the response of small samples collected from concrete buildings on a point-by-point basis. As a result, they are labor-intensive and time-consuming, and they cannot be applied uniformly across the building. Various non-destructive test (NDT) methods have been developed by several researchers that may be quickly applied to heat-damaged concrete in structures. Previous studies have highlighted the benefits and drawbacks of the nondestructive testing (NDT) methods routinely used to assess the state of heat-damaged concrete (see Table 1).

For the evaluation of the condition of heat-damaged concrete in the field and laboratory, NDT methods based on elastic wave velocity measurements (resonance vibration test, ultrasonic pulse velocity test, impact-echo, surface wave measurements, etc.) have gained popularity. The employment of ultrasonic pulse velocity (UPV) is the most common of these methods because of its simple operating principle, ease of equipment and data interpretation, and the fact that it is well-standardized in the test procedure in many countries across the world [19]. Choi et al. [20] studied and proposed two linear equations predicting the compressive and tensile strength of concrete using UPV. Kee et al. [7] investigated the effects of heating and cooling on the static and dynamic residual properties of 35 MPa concrete used for nuclear reactor auxiliary buildings in Korea and proposed correlations between static and dynamic properties of heat-damaged concrete. One study analyzed the effect of high temperatures on the residual property of concrete with fibers [21]. A similar study was performed on normal concrete (with a design strength of 50 MPa) and high-performance concrete (with a design strength of 98 MPa) [9]. In these studies, ultrasonic pulse velocities were usually correlated to the concrete mechanical properties. The traditional ultrasonic approach is sensitive to major faults or open fractures where there is an effective barrier to transmission, but not to equally dispersed microcracks or deterioration. Large defects in a specimen have a major impact on these linear parameters; therefore, monitoring the change in these linear parameters can help discover relatively large defects. Very minor faults, however, may have no effect on the linear parameters.

These minor faults, such as microcracks, can happen during the early fire exposure of concrete. Early fire damage in concrete usually happens because of its constituent materials such as binders, especially as it is one of the most important factors affecting the mechanical behavior of concrete exposed to high temperatures [22]. Beyond 100 °C, microcracks begin to develop in the hardened paste, and the hardened paste is subjected to an expansion-contraction cycle until it reaches 500 °C [23,24]. Choinska et al. [25] found that at 105 °C the pores in the porous concrete material enlarge due to heat expansion. When the temperature hits 150 °C, Portland cement paste constricted as the temperature rises. Thermal shrinkage of the cement paste and aggregate expansion cause thermal stresses in the cement pastes, resulting in micro-cracking in the concrete [26]. Dehydration, or the loss of non-evaporable water or hydration water, begins as temperatures reach 250 °C [27,28]. Between 200 °C and 250 °C, compressive strength begins to deteriorate significantly. The presence of microstructure in concrete does not necessarily affect the mechanical properties of concrete (e.g., compressive strength and modulus of elasticity) and the structural performance of reinforced concrete elements (ultimate strength and deformability). However, the behavior of microstructures can affect the permeability and durability properties of concrete structures, which could accelerate the deterioration of concrete from various sources. From the standpoint of infrastructure management agencies, special care is needed to monitor the condition of the concrete in structures that were partially or totally exposed to fire, and if necessary, to make appropriate maintenance actions. 

Nonlinear acoustical techniques might work in detecting early damage. There have been studies that used nonlinear UPV parameters such as side peak count [29,30,31], energy redistribution [30], coda wave interferometry [32], and the scaling subtracting method (SSM) [33] to assess the condition of concrete structures. Antonaci et al. [33] used the SSM to detect the delamination in concrete in the form of the discontinuity between two concrete cubes. Another study used nonlinear parameters in detecting surface-breaking cracks by utilizing the sideband energy redistribution and side peak count [29]. A study by Basu et al. [30] also used side peak count to monitor damage progression in reinforced concrete. In a study by Yim et al., a nonlinear ultrasonic method was used to estimate the compressive strength of thermally-damaged concrete [34]. Sun and Zhu used Coda wave interferometry to investigate the relative velocity changes of ultrasonic waves with temperature changes [35]. Yim et al. used an impact-modulation method to acquire nonlinearity parameters as a quantitative measure of contact-type defects [34]. Yang and Chen used a nonlinear ultrasonic second harmonic generation (SHG) technique to evaluate the degradation of concrete integrity exposed to extreme temperatures [36]. These nonlinear parameters are often calculated from some parts, usually near or at the tail-end, of the ultrasonic wave, which exhibits changes when the structure is damaged. Some of these studies, however, deal with measurements of surface waves, which restricts measurements due to wavelength scaling with propagating depth [36], and one study deals with temperatures of up to 200 °C only [35]. 

Another possible solution for exploiting ultrasonic waves to detect damage is the use of machine learning. Machine learning approaches using ultrasonic pulse data have been used in other fields. Starzak et al. [37] investigated the use of pulse velocity in patients with metabolic syndrome. Huang et al. [38] used CNN-LSTM to study damage detection in a copper pipeline using ultrasonic scanning. Machine learning technologies are increasingly being used in civil engineering due to the fast rise in data availability, as well as increased processing capacity and simpler programming methodologies. Daneshvar et al. [39] proposed a new approach for detecting and identifying damage by creating a new sensitivity function of modal strain energy. Another study by Daneshvar et al. [40] investigated the effects of impact loads brought on by the falling weight on heated-and-cooled reinforced concrete slabs using a multilayer perceptron (MLP). Research by Moradi et al. [41] predicted the compressive strength of concrete with binary supplementary cementitious material utilizing machine learning. Ultrasonic waves, much like vibration waves, are collected in the time domain. The development of machine learning for time-series analysis has been continuously improving. Machine learning has been used in some research to detect internal concrete deterioration using ultrasonic pulse data. Zhang et al. [42] used six different machine-learning approaches to estimate the degree of corrosion within rubber concrete. Karimpouli et al. [43] used machine learning in the ultrasonic prediction of crack density. According to the study of Hu et al. [44], the genetic algorithm backpropagation neural network (GA-BPNN), was effective in identifying pre-made voids inside concrete. However, while machine learning has been shown to be a reliable tool for detecting crack damage, there is still limited research on using machine learning with ultrasonic waves in detecting fire damage. Moreover, linear properties are the common parameters being used in these investigations, and there is still very limited research on nonlinear ultrasonic parameters of thermally-damaged concretes. The current study was established to investigate the use of the full waveforms from the ultrasonic signal measurements to make the most out of this parameter. Investigations on both the nonlinear parameters and the extracted parameters from the ultrasonic time series were also investigated in this research. 

This study aims to evaluate the damage of concrete exposed to high temperatures, especially focusing on the early detection of heat-induced damage. Ultrasonic pulse wave signals were used for the investigation. These signals were utilized to describe the effect of early thermal damage on both linear and nonlinear parameters of UPV. The same signals were also used to investigate the capability of using machine learning to classify concrete thermal damage. The data were gathered from cylindrical concrete specimens from three different design strengths exposed to six different target temperatures. The succeeding chapters discuss the methods used in sample preparation, data collection and preparation, the utilized machine learning methods, and network performance. The results in this study demonstrate the potential of machine learning of ultrasonic time series data for early detection of heat-induced concrete damage. 

## 2. Materials and Methods

**Figure 1:**
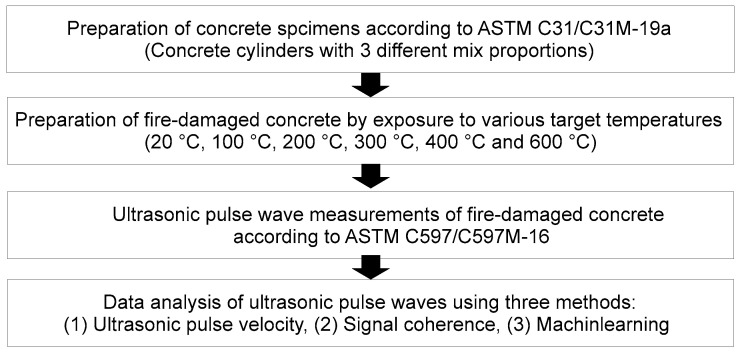
General experimental studies flow for this research.

This section describes the experimental methods used in the study to obtain the necessary time-series data for the analysis. In general, the study followed the flowchart shown in Figure 1. Concrete cylinder specimens were prepared and then subjected to different temperature levels to represent different degrees of thermal damage. After thermal exposure, ultrasonic pulse waves were measured.

### 2.1. Sample Preparation and Exposure to High Temperature

Sample concrete cylinders (200 mm height × 100 mm diameter) were fabricated for use in all the tests that were done for the study. Three concrete mixes were used, with different water-to-binder ratios—Mix 1, Mix 2, and Mix 3. The properties and quantities of the samples are presented in Table 2. Thirty-five concrete cylinders were cast in 100 mm by 200 mm plastic molds in accordance with ASTM C31/C31M [45] for each concrete mix. Thirty cylinders (i.e., five cylinders per each target temperature) were used to evaluate the thermal properties of concrete after heating and cooling with six target temperatures (20 °C for control specimens, and 100 °C, 200 °C, 300 °C, 400 °C, and 600 °C). In addition, ten concrete cylinders were prepared to monitor the internal temperature of concrete during the heating and cooling process for the test specimens. For these specimens, a plastic tube with a diameter of 0.5 mm was fitted inside the concrete specimen. The tube served as a casing for the placement of a thermocouple inside the specimen. All concrete cylinders were water-cured in a water tank with a constant temperature (20 °C) after being de-molded on the day after they were cast. Six concrete cylinders (five test specimens and one for measurements of the core temperature of the concrete) were taken from the water tank and dried in an electric oven at 100 °C. After 24 h, heating was stopped to let the specimens cool naturally back to room temperature (20 °C~23 °C) and the duration of the cooling process was referenced to the temperature of the oven. This process was done for all concrete cylinders before they were exposed to the target temperature inside the furnace. This added step was done to ensure that all specimens had a consistent water content within 1% at the start of the concrete ‘firing’. It is widely known that continuous drying of concrete at 100 °C only results in the evaporation of the physical free water without causing thermal damage associated with dehydration and decomposition of the hydrates in concrete [46,47]. Therefore, the concrete specimens after the oven-drying and cooling process were defined as “without fire damage” in this study. 

**Figure 2:**
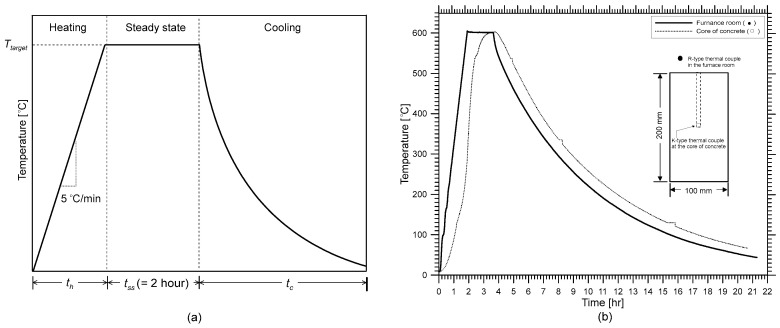
Temperature-time history used for this research: (**a**) programmed temperature-time history for heating electric furnace, (**b**) measured temperature in the air of the furnace and the core of a concrete cylinder for the target temperature of 600 °C. Note that *t_h_* is defined as the time it takes to reach the target temperature, *t_ss_* is the time when the temperature is sustained at *T_target_* (target temperature for this study, lasts for 2 h), and *t_c_* is the time it takes to naturally cool down to room temperature.

The concrete cylinders were heated in a programmed electric furnace with a temperature history control shown in Figure 2a. An R-type thermocouple (platinum and platinum-rhodium alloy, 87 percent Pt and 13 percent Rh by weight) was installed in the electric furnace to monitor air temperature. The electric furnace heated six concrete specimens at the same time. A K-type thermocouple (chromel and alumel alloy) was buried in one of the concrete cylinders to measure the core temperature of the concrete. Note that the R-type thermocouple gives stable and accurate (±1.5 °C) results and is used in high-temperature applications (0 °C to 1600 °C) [48], but it is relatively expensive; the K-type thermocouple is moderately accurate (2.2 °C), can be used in a wide temperature range (0 °C to 1260 °C) [48], and is cost-effective for measuring concrete temperature. The heating-cooling method was patterned after the previous literature [7,22,49,50] with a heating rate of 5°C per minute. The six concrete cylinders were placed inside the electric furnace for exposure to five different temperature levels (100 °C, 200 °C, 300 °C, 400 °C, or 600 °C) and room temperature (20 °C) for control data. The temperature of the electric furnace was held constant around the goal temperature in this investigation until the concrete’s core temperature approached thermally steady-state conditions. The steady-state condition was defined as the temperature at which the concrete’s core temperature was equal to its surface temperature, or when the internal temperature increment rate was less than 0.5 °C/min. Figure 2b shows the measured temperature-time history of the internal temperature of the furnace and core of the concrete cylinder for the target temperature of 600 °C. It was observed that the measured core temperature in the concrete cylinder approached the thermally steady-state condition after the internal temperature of the furnace was sustained at the target temperature for about 2 h. The electric furnace’s heat source was shut off after the temperature of the cylinders reached a steady-state, and the furnace was naturally cooled in an airtight state. During the cooling period, the temperature of the concrete decreases according to an exponential function [51] (T = c_0_e^γt^, where c_0_ is a constant dependent on a target temperature in °C; γ is a cooling rate of about −0.14/h for all concrete specimens and target temperature levels; *T* is the concrete temperature, and *t* is time in hours).

### 2.2. Ultrasonic Pulse Wave Measurements

**Figure 3:**
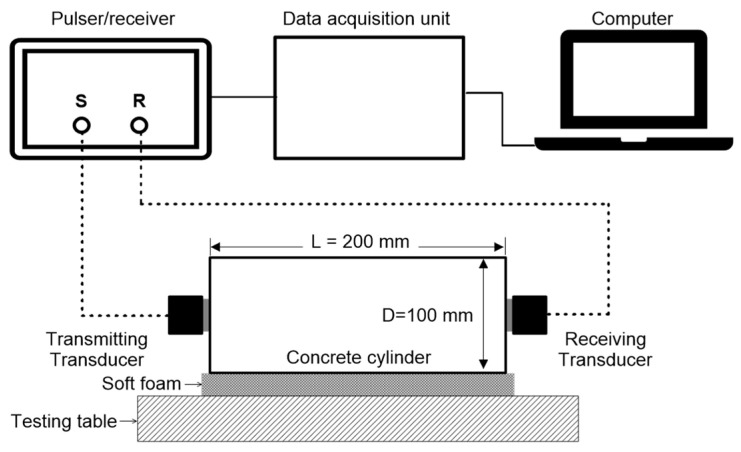
Test setup for ultrasonic pulse velocity measurements.

Figure 3 illustrates the test setup of ultrasonic pulse wave measurements transmitted through a concrete sample. The standard test procedure according to ASTM C 597/C597M-16 [52] was used to assess the P-wave velocity (constrained compressive velocity) of concrete cylinders that were subjected to different target temperatures. The room temperature during the ultrasonic pulse wave measurements was maintained at around 20 °C to 25 °C throughout the experiment. The study used a pair of transducers with a center frequency of about 50 kHz that can transmit and receive ultrasonic pulses (see Figure 3). Using a pulse-receiver (Panametrics 5077 PR, Tokyo, Japan), a 200 V square pulse with a duration of 10 µs was used to drive the source transducer (Olympus, Tokyo, Japan). First, the concrete surface was wiped with a wet cloth to remove dust. Next, the standard coupling agent was applied on the contact surfaces of the concrete and transducers to minimize impedance mismatch in the ultrasonic pulse wave measurements. Furthermore, the transducers were firmly pressed to minimize the air gaps in the concrete and transducer interfaces. The receiving sensor recorded transient stress waves that were created by the source sensor and propagated through the concrete. It took a couple of seconds to obtain stable signals after starting the ultrasonic pulse wave measurements. The receiving signals were digitized by a high-speed digital oscilloscope (NI-PXI 5101, Austin, TX, USA) with a total signal length of 0.001 s at a sampling rate of 10 MHz after the measured ultrasonic signals became stable. In this study, pulse data were collected ten times on each cylinder and were averaged to improve signal-to-noise levels of the measured data. The averaged ultrasonic data was transferred to a laptop computer for data storage and post-processing. 

**Figure 4:**
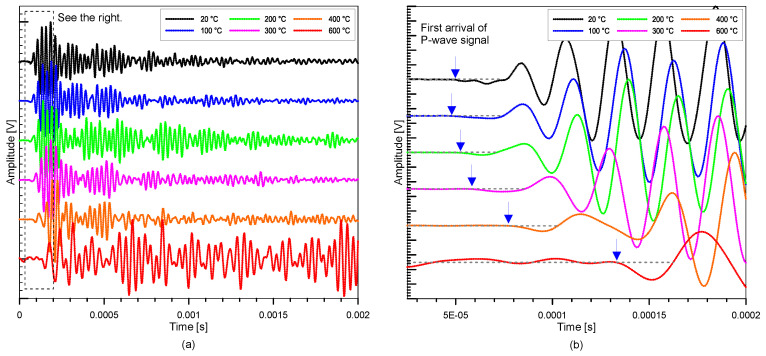
Typical time signals of ultrasonic pulse waves propagating through the thermally-damaged concrete cylinders under different target temperatures: (**a**) ultrasonic waves at different target temperatures and (**b**) enlarged ultrasonic signal around the first arrival of the wave shown in (**a**).

Figure 4a presents the typical P-wave signals measured from the concrete cylinders used in this study, and the enlarged signals are shown in Figure 4b. The signals from Figure 4a,b were processed in MATLAB [53] using the ‘smoothdata’ function for smoothing the signals and normalization by dividing the whole signal function by its maximum value. The velocity of an ultrasonic wave can be calculated by dividing the wave path, *L*, by the travel time, *t*, as follows:

(1) $$V_{p}=\frac{L}{t_{a}-t_{d}}$$

where *V_p_* is the wave propagation velocity, *L* is the distance between transducers, *t_a_* is the initial wave arrival time, and *t_d_* is the delay time computed during probe calibration. When the two transducers were positioned opposite each other, the time for the first arrival wave was recorded, and the delay time was calculated. It should be noted that P-waves are potentially faster in time signals than any other refracted and reflected waves from the border of concrete cylinders. The arrival of transient stress waves via cylinders was computed using the modified threshold approach [54] based on the observed ultrasonic signals. Using the conventional threshold method used in earlier investigations [55], an estimated arrival time was initially obtained in this way. After that, a precise arrival time was calculated by fitting a line to the signal data. The intersection of the two P-wave travel times was then used to determine the P-wave travel time. The intersection of the fitting line and the measured zero-signal stage was used to determine the P-wave travel time. However, more care was needed to find the first arrival time of the P-wave in the ultrasonic pulse waves from the severely damaged concrete after being exposed to 600 °C. In this study, a low pass filter, with a cut-off frequency of 30 kHz, was applied to suppress high-frequency noises that appeared before the arrival of the P-wave.

To analyze the nonlinear parameters of ultrasonic wave signals, this study used signal coherence. Coherence is a statistical metric that measures the degree of correlation between two signals as a function of frequency. Some research studies have related coherence to microelectronics engineering [56,57], modal analysis [58,59,60,61], and signal processing [62,63]. The coherence function has been employed in concrete investigations; however, its use is currently confined to ensuring signal consistency between ultrasonic signal measurements [10,64]. The presence of out-of-phase noise, irregularity of coupling between the source and receiving transducers during testing, and the change of internal configuration due to defects and voids are the key variables that impair coherence [10]. The coherence function is calculated by

(2) $$\gamma_{xy}\left(f\right)=\frac{S_{xy}\left(f\right)}{\sqrt{S_{xx}\left(f\right)S_{yy}\left(f\right)}}$$

where $\gamma_{xy}\left(f\right)$ is the coherence, $S_{xy}\left(f\right)$ is the cross-spectral density of *x* and *y*, and $S_{xx}\left(f\right)$ and $\;S_{yy}\left(f\right)$ are the power spectral densities of *x* and *y*, respectively. The resultant value is a number between 0 and 1.0, with a value around 1.0 indicating high signal coherence.

**Figure 5:**
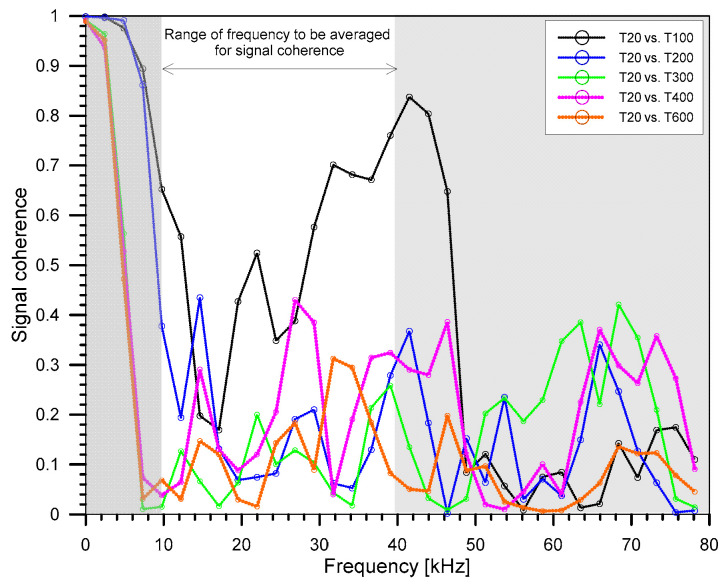
Frequency window for averaging magnitude squared coherence (MSC).

As can be seen from the equation, assessing the coherence between concretes with and without fire damage requires a baseline value. To obtain the coherence values in this study, signals were initially collected on specimens with and without fire damage. A subset of the time-domain signals was then chosen, and each signal was converted into power spectral density using FFT. The coherence was computed from the converted signals using MATLAB’s ‘mscohere’ function [53]. The coherence value is averaged within a certain frequency frame in this investigation so that the outcome may be reported as a single number and analyzed with temperature variations. In this study, the tail end of the ultrasonic wave signal was used (shown in Figure 4 as the signal enclosed inside the dashed box). Figure 5 illustrates the frequency used for this study in averaging the coherence of the time signals from the concrete. The primary wave going through the concrete is captured in the first half of the signal. The reflected signal towards the tail end of the transmission, however, has a lower amplitude because of energy absorption. So far, there are still no standards on what ‘window’ should be used for averaging nonlinear properties. Therefore, the coherence averaging approach, like other nonlinear methods, still clearly requires engineering judgment. For this analysis, the ultrasonic signals from the different temperatures were each compared to the signals from the normal temperature condition (20 °C vs. 100 °C, 20 °C vs. 200 °C, 20 °C vs. 300 °C, 20 °C vs. 400 °C, and 20 °C vs. 600 °C).

### 2.3. Machine Learning for Full Wave Analysis

#### 2.3.1. Preprocessing of Time-Series Data from UPV Measurement

**Figure 6:**
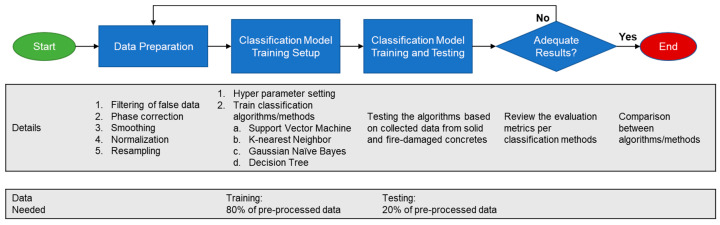
General flowchart for the machine learning method used in this study.

Before machine learning classification, it is important that the time signals from the UPV measurement were first preprocessed. For the preprocessing of the time series, the study followed the methodology presented in Figure 6. 

Eighty-three concrete cylinders were exposed to thermal damage. Each cylinder was then assessed for the UPV wave measurements. For UPV measurements, 10 readings were taken for each cylinder giving a total of 830 readings. Since the 10 readings represent one concrete specimen under the same temperature condition, the average of 10 signals was used to represent a specimen under a specific condition. This procedure was done after checking the coherence of the 10 readings that were taken for the same specimen on each temperature condition. In all, only 83 sample data were considered for the machine learning classification system. Unfortunately, due to time, access, or interpretability restrictions, the number of datasets considered in this study may not be sufficient to develop a general model. Nevertheless, the number of samples in this study is more than 10 times greater than the temperature levels (20 °C, 100 °C, 200 °C, 300 °C, 400 °C, and 600 °C) considered in the classification models in this study, which is comparable with ‘a rule of thumb’ of the required training datasets for a machine leaning process [65]. Therefore, the number of samples (=83 sample data) in this study can be said to be acceptable to investigate the feasibility of machine learning of ultrasonic pulse waves to evaluate early heat-induced concrete damage [65].

Filtering erroneous data was the first step in the preparation procedure. Imperfect coupling can cause erroneous data, leading to a signal with non-sinusoidal shapes. Signals that were either excessively loud or too weak, as indicated by signal clipping or signal-to-noise indistinguishability, were deleted as well. Furthermore, due to a hardware constraint, some received signal data included a high amplitude signal in the same time step as the source or ‘ghost’ signal. The next step is adjusting the signal to the correct phase. Some signals have delays in them due to the constraint of the oscilloscope. The phase of some signals might be delayed but their integrity is still retained and they can still be considered an ‘acceptable’ signal. The time window for each signal was then reduced to 7.5 ms to compensate for the loss of some data in the adjusted signal. Smoothing and normalizing the time signals were then done so that all signals had a consistent amplitude. The data was smoothed by computing local quadratic equations in each window of the data being analyzed. This method results in fewer discontinuities but requires more computation. As for the normalization of data, the study followed the conventional method in MATLAB [53] where the values of the peak amplitudes range between −1 to 1. Using this method, the signal’s amplitude is scaled over all samples so that the peak magnitude is set to 1. Data resampling was then done to consider different sampling rates for the analysis. Instead of depending on a model based on a predetermined equation, machine learning algorithms use computer methods to “learn” information directly from data. The algorithms modify their performance as the number of examples available for learning increases. 

For machine learning classification, two sampling rates were used—10 MHz, which is the default sampling rate of the equipment used, and 125 kHz. The data for the UPV time signals were preprocessed to reflect the 125 kHz sampling rate. In terms of time windows, the study used lengths of 9 ms (for comparison of the two sampling rates used), 7.5 ms, 5.5 ms, 3.5 ms, and 1.5 ms. There would be six classification groups for each target temperature (20 °C, 100 °C, 200 °C, 300 °C, 400 °C, and 600 °C) labeled as 0, 1, 2, 3, 4, and 5, respectively. For this analysis, this study used the Classification Learner application built into MATLAB [53].

#### 2.3.2. Classification Algorithms

In this study, machine learning was used to classify the thermal damage of the concrete specimens that were exposed to different temperatures. The different machine-learning methods used for this study are summarized in Table 3. The data used for the analysis was from the ultrasonic waves of the UPV measurement. 

Support vector machine (SVM) for classification is a supervised learning technique. This method has been used in some studies for time-series prediction or forecasting [56,66,67]. A support vector machine, in more formal terms, creates a hyperplane or set of hyperplanes in a high- or infinite-dimensional space that can be used for classification and regression in a variety of activities such as image retrials, financial study, and so on. The idea behind SVM is to use the “hyperplane” as a decision boundary (see Equation (3)), to divide learning targets into positive and negative classes, and to make any sample’s point-to-plane distance (see Equation (3)) larger than or equal to 1.

(3) $$w^{T}x+b=0$$

(4) $$y_{i}\;\left(w^{T}\;x_{i}+b\right)\;\geq \;1$$

K-nearest neighbor (KNN) is a non-parametric supervised learning method. The function is only approximated locally in k-NN classification, and all computation is postponed until the function is evaluated. Because this method relies on distance for classification, normalizing the training data can greatly increase its performance if the features represent various physical units or come in wildly different scales. It provides a high level of performance [68]. The KNN classifier has a few disadvantages, including a long execution time, sensitivity to large datasets, sensitivity to the value of parameter K, and a large number of computing steps [69]. K-nearest neighbor is a classic learning strategy in which no model is built from the training data. A distance function, such as the Euclidean or Manhattan distance metric, is employed to assess the similarity of an unknown instance to each instance in the training set. The unknown instance’s class label is then chosen by a majority vote among its K closest neighbors. The theoretical features of the KNN rule ensure that its probability of error is bounded above twice the Bayes probability of error for any distributions.

Naive Bayes classification models are widely used for a variety of real-world tasks, including email spam filtering, text categorization, and document classification. This family of probability classifiers is based on a very simple premise, and they perform extremely well in training and prediction on high-dimensional datasets. They just require a modest amount of training data to calculate the required parameters, and their findings are usually always interpretable, unlike neural networks, where this is frequently impossible [70]. In general, naive Bayes classifiers use Bayes’ theorem to apply a strong mutually independency (naive) assumption between features. This means that the probability p(y_k_|v_1_, …, v_N_) of a given voltage value v_i_ (feature) belonging to a given class value (also called label or target value) y_k_, i.e., being a correct (k = +) or false (k = −) pulse, is independent of all other (N − 1) voltages representing the detector pulse v = {v_1_, …, v_N_} of length N. (feature vector).

A Decision Tree (DT) is a flowchart-like structure model that represents its findings using a tree-like structure made up of internal nodes that carry test conditions and class labels or leaf nodes (decision made after computing all features). The DT method has a built-in feature selection mechanism since it assesses the importance of the attribute when creating test conditions [71,72]. Because of these qualities, as well as its predictive power, DT is one of the most extensively used classifiers. 

#### 2.3.3. Input Data

The types of input data for the machine learning process were also considered for this study. As discussed in the previous section, the sampling rate and the time window of the signals were also investigated. The preprocessed time series or signal was used to compare the different sampling rates and different time windows. All signals were adjusted to reflect the source signal, i.e., time zero (*t*_0_) is at the beginning of the source signal. As discussed in Section 2.3.1, above, the time window was adjusted to compensate for the loss of other signal data due to preprocessing, so the length signal was reduced to 9 ms.

Feature extraction from the time series was also considered for use for inputting data in machine learning. In one study by Li et al. [73], the researchers observed that extracting some features from the time series can improve the model accuracy for machine learning or deep networks. For this study, the two features extracted were spectral entropy and instantaneous frequency. Spectral entropy is based on Shannon entropy in the field of information theory, which measures the spectral power distribution of the data *x* with N number of samples in different frequencies. The fundamental equations are as given in [74].

Typically, the part of the time series that contains the signal will have a higher entropy than another part that is purely noise. The instantaneous or soon-to-be frequency is a measure of the change in the time parameter of a nonstationary signal associated with the average of the frequencies as the signal alters. More details regarding instantaneous frequency can be found in the research by Boashash [75].

#### 2.3.4. Training and Testing

Matlab R2022a [53] Classification Learner toolbox was used to train and test the model. The hyperparameters used were set as default in the toolbox. The hyperparameters for training the network were set up once the data had been prepared. For validation of the algorithms, k-Fold cross-validation was used with the value of k-folds set to the default value of five. This means that the dataset was shuffled and then split into 5 groups with each group having 16 observations. The selected algorithm would then be trained and evaluated with each group, i.e., the model or algorithm would train on four of the five groups and then be evaluated or tested on the remaining group.

For each algorithm used in machine learning, the dataset was prepared with the same preprocessing method as discussed in Section 2.3.1. Afterward, default parameters were assigned in the MATLAB [53] Classification Learner Toolbox. For the SVM algorithm, the linear Kernel function was used with a Kernel box constraint level and Kernel scale both equal to 1 as default values. For this algorithm, the multiclass method used was ‘One-vs-One’. For the KNN algorithm, the number of neighbors was set to three with a distance metric of Euclidian and distance weight set to ‘Equal’. For the Naïve Bayes algorithm, the default hyperparameters set by MATLAB [53] were used for the Gaussian kernel. For the Decision Tree algorithm, the maximum number of splits used was 100 with the split criterion set to ‘Gini’s diversity index’. All misclassification cost was set to default.

#### 2.3.5. Performance Measure

**Figure 7:**
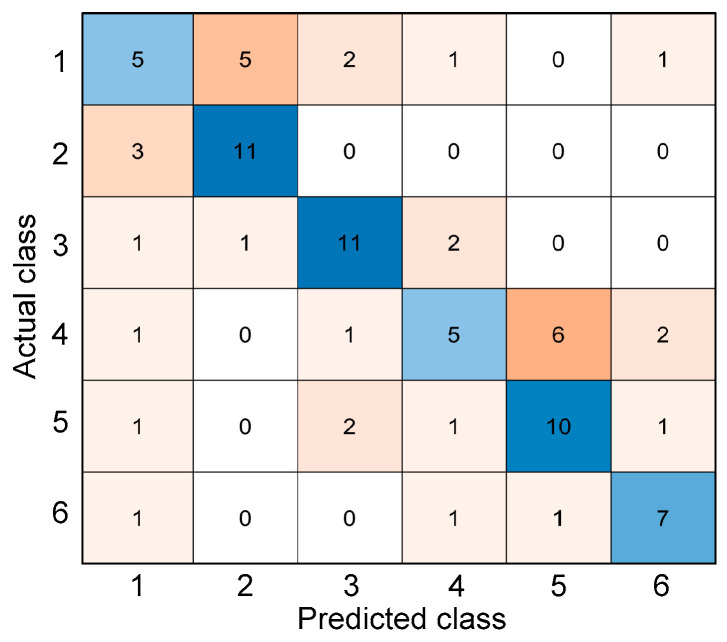
Example of a confusion matrix.

There are many evaluation metrics used to investigate the performance of a machine learning classification. These metrics are often related to the confusion matrix, which is a specialized table layout that visualizes the performance of a supervised learning algorithm. It was named as such to demonstrate whether the algorithm is confusing different classes, i.e., mislabeling a class as another class. This table has two dimensions—actual and predicted—with equal sets of classes in each dimension. An example of a confusion matrix can be seen in Figure 7. The blue color represents the true positives—correctly predicted classes—while the other colors represent the mislabels or misclassifications. The intensity of the color depends on the number of inputs that were labeled in a particular class, i.e., darkest blue represents the highest number of inputs that were correctly labeled while the darkest orange represents those that were incorrectly labeled.

From this table, different evaluation metrics can be calculated. The basic metrics are accuracy, *F*1-score, precision, and recall. Accuracy describes how the model performs in classifying the time signals. Precision is the ratio of correctly predicted positive observations to the total predicted positive observations, while recall is the ratio of correctly predicted positive values to all observations in that particular class. Precision and recall exist in a trade-off relationship, i.e., optimizing one comes at the cost of the other. To avoid this, *F*1-score is used, which utilizes precision and recall to present the test’s accuracy through a harmonic mean. Theses metrics can be calculated using the following equations:

(5) $$Accuracy=\frac{\left(TP+TN\right)}{\left(TP+TN+FP+FN\right)}$$

(6) $$Precision=\frac{TP}{TP+FP}$$

(7) $$Recall=\frac{TP}{TP+FN}$$

(8) $$F1-score=\frac{2\times (Precision\times Recall)}{Precision+Recall}$$

where *TP* is true positives (correctly predicted positive classes), *TN* is true negatives (correctly predicted negative classes), *FP* is false positives (incorrectly predicted positive classes), and *FN* is false negatives (incorrectly predicted negative classes). For this study, accuracy and *F*1-*score* were used to evaluate the performance of the machine learning algorithms.

## 3. Results and Discussion

### 3.1. Ultrasonic Pulse Velocity

**Figure 8:**
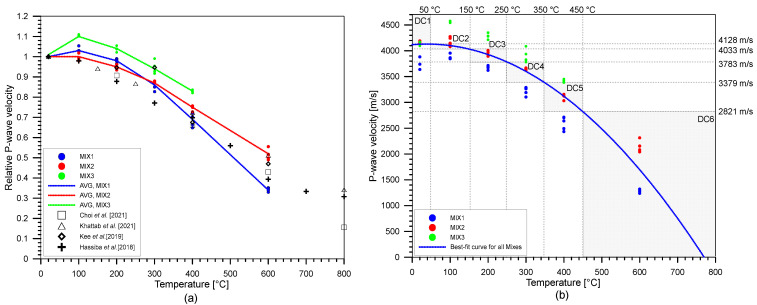
Variation of (**a**) relative P-wave velocities $\left(\frac{V_{p_{T}}}{V_{P20}}\right)$ from the experimental data of this study and the literature with increasing temperature and (**b**) labeled P-wave velocities. Note that DC in (**b**) means damage class of concrete.

**Figure 9:**
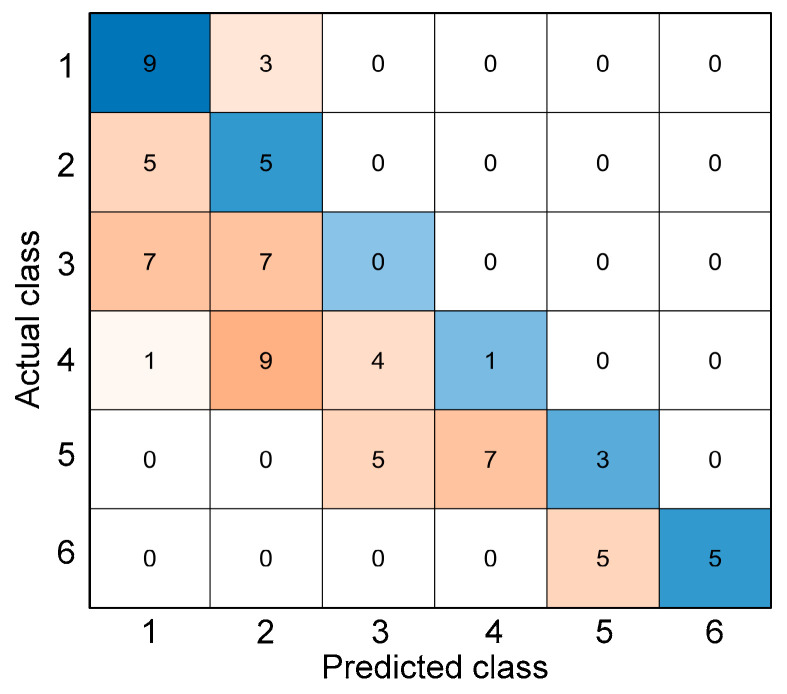
Confusion matrix from classifying the temperatures derived from the regression equations used from P-wave velocities.

Figure 8 shows the variation of the ultrasonic pulse wave velocity, *V_p_*, of heat-damaged concrete with increasing temperature. The results from the concrete for the Mixes 1, 2, and 3 are shown as red, blue, and black solid circles. The average value of the P-wave velocity ranges from 3019.20 m/s to 4029.11 m/s across different mix designs and different temperatures. The COV varies from 4.68% to 26.92% with the highest COV found with the temperature of 600 °C. For all mixes of concrete, there is a slight increase of wave velocity from room temperature to 100 °C, then the wave velocity dropped at 200 °C. Between 20 °C and 100 °C, the wave velocity increased to about 3.3% for Mix 1, 0.1% for Mix 2, and 10.4% for Mix 3, of the wave velocity at room temperature. At 200 °C, the P-wave velocity dropped to about 2.0% of the *V_p_* measured at 20 °C, *V_P20_*, for Mix 1 and 5.4% of *V_P20_* for Mix 2. Conversely, the wave velocity of Mix 3 decreased from 100 °C to 200 °C but was still higher than *V_P20_* by about 3.8%. By 300 °C, the decrease of P-wave velocities was apparent, dropping by around 14.1%, 12.6%, and 5.7% for Mix 1, Mix 2, and Mix 3, respectively. Furthermore, by 400 °C and 600 °C, P-wave velocities of all mixes substantially dropped to around 17.0% to 65.8% of *V_P20_*, with Mix 1 having the highest percentage drop, equal to 65.8% after being exposed to 600 °C. Results from the literature [7,21,49,76] are also shown to evaluate the results from this work. As expected, the effect of the thermal exposure cannot be clearly seen because of a minimal change in the P-wave velocity values with varying the exposure temperature from 20 °C to 300 °C. Other studies [21,76] also show that there is a minimal change in the values of the P-wave velocities from room temperature to, at most, 300 °C. In this study, a nonlinear equation was used to relate the exposure temperature and P-wave velocity of heat-damaged concrete as follows,

(9) $$V_{P}=4119-0.0077T_{}^{2}+0.5817T,\mathrm{for}\;37.8\;{}^{\circ}C\leq T\leq 770\;{}^{\circ}C$$

where *V_p_* is the P-wave velocity in m/s and *T* is the exposure temperature in °C. The use of a linear parameter of the ultrasonic wave showed that exposure of concrete cylinders to relatively low temperatures did not affect the P-wave velocities significantly. It can be seen from the graph that there is a gradual decrease in the P-wave velocity in the early exposure to fire, until the temperature of 300 °C. From this temperature until 600 °C, a sudden decrease can be observed. Values of P-wave velocity are also fluctuating between the range of 20 °C to 300 °C. For this parameter, six classes were also established from the ranges of temperatures—class 1 for P-wave velocities when the temperature is T < 50 °C (*V_p_* > 4128 m/s), class 2 for 50 °C ≤ T < 150 °C (4033 m/s < *V_p_* ≤ 4128 m/s), class 3 for 150 °C ≤ T < 250 °C (3783 m/s < *V_p_* ≤ 4033 m/s), class 4 for 250 °C ≤ T < 350 °C (3379 m/s < *V_p_* ≤ 3783 m/s), class 5 for 350 °C ≤ T < 450 °C (2821 m/s < *V_p_* ≤ 3379 m/s), and class 6 for T ≥ 450 °C (*V_p_* ≤ 2821 m/s). This classification system is also visualized in Figure 8b. Figure 9 shows the confusion matrix from the results of classifying after using Equation (9) in predicting the corresponding temperature and consequently classifying to specific classes. This method of using the equations derived from the P-wave velocities of the experimental data yielded an accuracy of 30.26%. It can be seen that the relatively low accuracy is caused by fluctuating trends of P-wave velocity measurements, especially for concrete specimens exposed at lower temperatures (≤300 °C).

### 3.2. Coherence of Ultrasonic Pulse Waves

**Figure 10:**
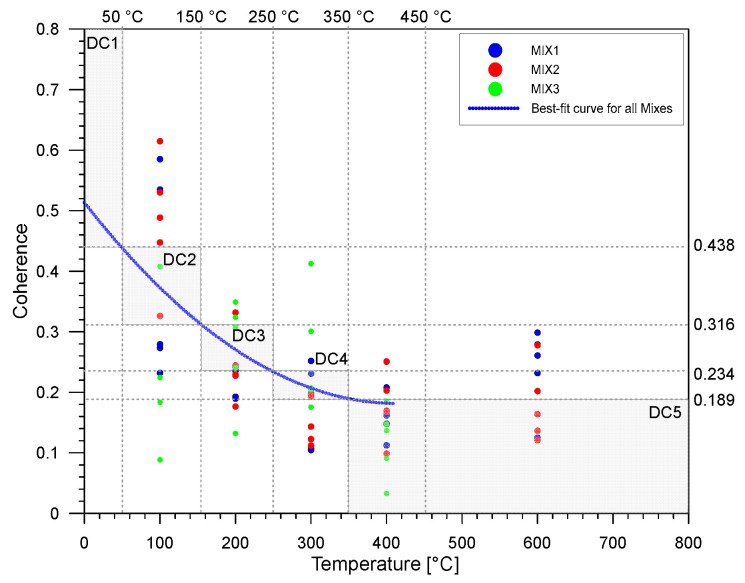
Variation of coherence with increasing temperatures and their corresponding classes. Note that DC means damage class of concrete.

Figure 10 presents the coherence values of the ultrasonic signals from concrete specimens from different temperatures with the specimens exposed to the room temperature condition (=20 °C in this study). The average value of coherence ranges from 0.155 to 0.373 across different mix designs and different temperatures. The COV varies from 25.93% to 44.29% with the highest COV found with the temperature of 600 °C. The coherence between the ultrasonic wave signals was compared against the condition under the normal temperature condition. The difference in averaged signal coherence is most noticeable when the signal is studied on the tail end part of the signal and averaged between 10 kHz to 40 kHz. In general, at least for Mixes 1 and 2, the coherence between the signals was high between 20 °C and 100 °C with values equal to 0.541 and 0.611, respectively. However, for the remaining temperature conditions, the values decreased considerably but still fluctuated with values in the range of 0.110 to 0.223. As for Mix 3, the results for the coherence are inconclusive. The low average coherence value for non-fire-damaged specimens suggests that the signal is more dispersed inside the specimen, which could be attributed to the formation of cracks and voids as the temperature increases. Signals from fire-damaged specimens will have a relatively low coherence compared to signals from solid specimens, which have less scattering due to the lack of voids or fissures. 

**Figure 11:**
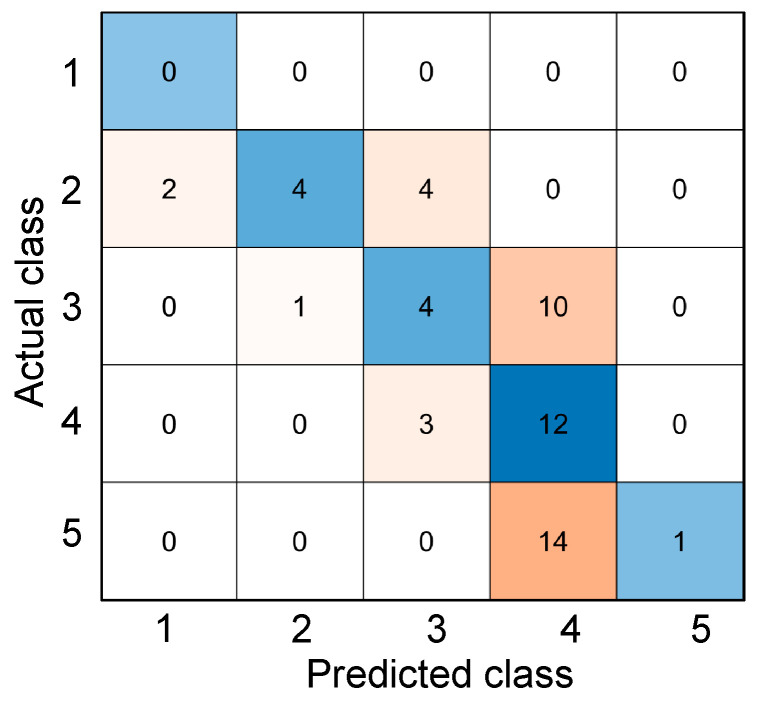
Confusion matrix from classifying the temperatures derived from the regression equations used from coherence values.

From these results and observations, it can be assumed that nonlinear UPV parameters have the potential to be used for analyzing data collected from fire-damaged concrete. In contrast with P-wave velocity, the use of coherence exhibited good detection of differences in parameter values between the exposure to room temperature and lower elevated temperatures. There is a clear decrease in coherence in this range of temperatures. However, this observation is only true until 200 °C. There are fluctuating values between coherence after this temperature. In this study, an approximate formula that relates the coherence and the corresponding temperature was established by a non-linear regression analysis as follows,

(10) $$coh=0.5135+1.928\times 10^{-6}T^{2}-0.0016T\;\;\;\mathrm{for}\;\;\;0\;{}^{\circ}C\;\leq \;T\leq \;410\;{}^{\circ}C$$

where *coh* is the coherence value and *T* is temperature. Equation (10) was used to classify the specific condition class of fire-damaged concrete cylinders based on the coherence measurements. For this parameter, six classes were also established from the ranges of temperatures—class 1 for coherence when the temperature is T < 50 °C (*coh* > 0.438), class 2 for 50 °C ≤ T < 150 °C (0.316 < *coh* ≤ 0.438), class 3 for 150 °C ≤ T < 250 °C (0.234 < *coh* ≤ 0.316), class 4 for 250 °C ≤ T < 350 °C (0.189 < *coh* ≤ 0.234), class 5 for 350 °C ≤ T (*coh* ≤ 0.189). This classification system is also visualized in Figure 10. Figure 11 shows the confusion matrix from the results of classifying after using Equation (10) in predicting the corresponding temperature and consequently classifying to specific classes. This method of using the equations derived from the coherence values of the experimental data yielded an accuracy of 32.31%. From this figure, it can be observed that the coherence values of concrete specimens exposed at any temperature still give fluctuating values of predicted temperature. 

### 3.3. Machine Learning of Ultrasonic Pulse Waves

#### 3.3.1. Effect of Preprocessing Parameters

It is important to determine the appropriate data format for the input layer of the machining learning process. This section compares the performance of machine learning with some important parameters for preprocessing of ultrasonic pulse waves obtained from thermally-damaged concrete with various severity levels. The main parameters considered in this study include the time window (signal length), the sampling rate of signals, and types of input data (time series signal, spectral entropy, and instantaneous frequency). In this study, accuracy was used as a performance measure of the classification models. 

**Figure 12:**
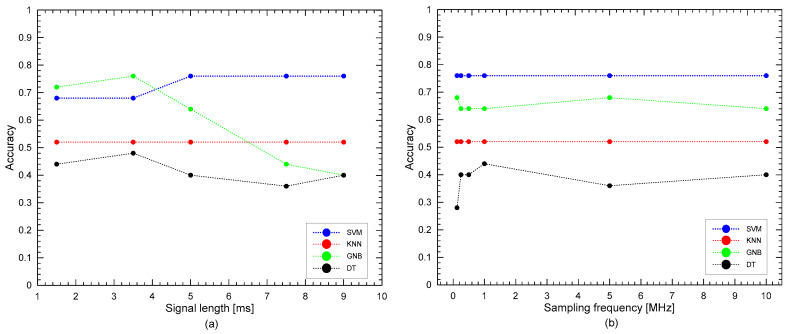
Comparison of accuracies using different variables from preprocessing: (**a**) signal length (with sampling rate of 10 MHz), (**b**) sampling rate frequency (with signal length of 5 ms).

Figure 12a shows the comparison of the accuracy using time series with various signal lengths as the input of machine learning. For this analysis, the tail end of the signal was cut off. This is also done to minimize the noise in the signal which is normally found at the tail end. Five different signal lengths were considered in this study, 9 ms, 7.5 ms, 5 ms, 3.5 ms, and 1.5 ms. The length of the time signal dictates the number of features, or data points, to be included in training the classification model. In this study, time series with a length of 9 ms gives 90,000 features, 7.5 ms gives 75,000 features, 5 ms gives 50,000 features, 3.5 ms gives 35,000 features, and 1.5 ms gives 15,000 features. The sampling frequency of the time series was constant at 10 MHz, the default value from the laboratory equipment used in this study. As can be seen from the graph, signal length had a different effect on the accuracy of different models. For SVM and KNN, the accuracy of the model was stable, at 76% and 52%, regardless of the signal lengths used, until the signal length of 5 ms, and then suddenly decreases to 64% and 52% as the length decreased to 0.75 ms and 0.3 ms, respectively. The results show that the length of ultrasonic signals should be sufficiently long to ensure the stable performance of SVM and KNN. It can be inferred that the later part of the ultrasonic signals includes useful information for characterizing early damage of concrete exposed to fire. In contrast, the use of GNB and DT resulted in the best accuracy when the signal length was 3.5 ms. For the machine learning of ultrasonic pulse waves from fire-damage concrete cylinders, a signal length of 5.0 ms was determined to be optimal for the SVM and KNN, whereas for GNB and DT, the optimal signal length was determined to be 3.5 ms. 

Figure 12b shows the comparison of accuracy using time series with various sampling rates. A total of six different sampling rates were considered for the analysis: 10 MHz, which is the default sampling rate of the equipment used, 5 MHz, 1 MHz, 500 kHz, 250 kHz, and 125 kHz. The ultrasonic pulse wave signals were preprocessed to reflect the lower sampling rates. This study used time series with lengths of 5 ms and 3.5 ms as discussed in the preceding section. As can be seen from the graph, the accuracy of the model did not significantly change when using different sampling rates for SVM and KNN for both time windows used, with an accuracy equal to 76%. From these interpretations, it could be said that using even the lowest sampling rate of 125 kHz is enough for analyzing time signals in machine learning to classify concrete specimens for fire damage. This result is informative on the selection of optimum devices for collecting ultrasonic pulse waves for machine learning. 

**Figure 13:**
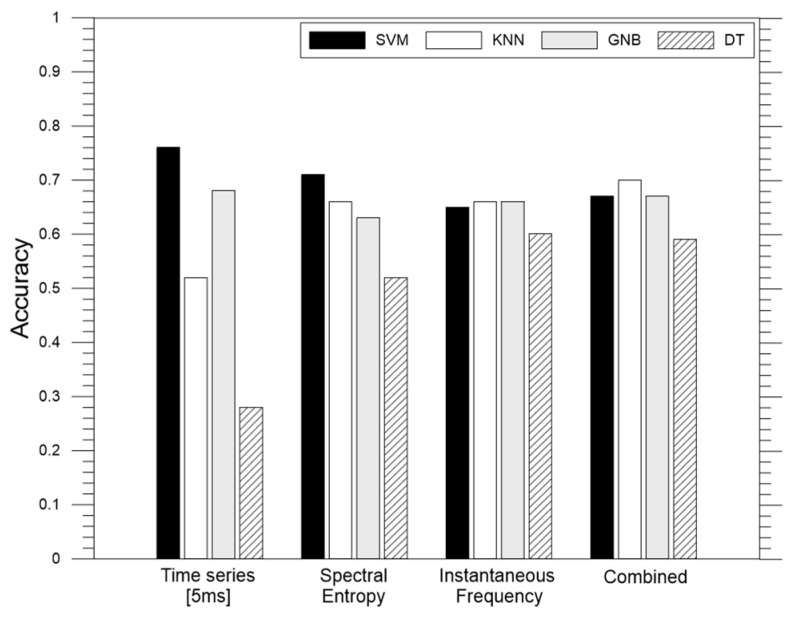
Comparison of accuracies using different input data from preprocessing. Note that the sampling rate used is 125 kHz and ‘Combined’ represents the combination of spectral entropy and instantaneous frequency.

In addition, the effect of the use of frequency domain signals (e.g., spectral entropy and instantaneous frequency) was investigated to find the optimum data format for the input of machine learning. Figure 13 shows the comparison of the overall accuracy of using three different combinations of frequency domain signals (spectral entropy only, instantaneous frequency only, and a combination of both) along with four different machine leaning algorithms. The frequency domain signals were obtained from the fast Fourier transform of the time series with a sampling rate of 125 kHz and signal length of 5 ms. The average overall accuracy of using the combination of spectral entropy and instantaneous frequency is 65.96% while that of using spectral entropy only is 62.95%, and that of using instantaneous frequency only is 64.46%. Overall, the use of spectral entropy and instantaneous frequency did not significantly improve the accuracy of the classification of the fire damage compared to using time series data as the input of machine learning. To recall, the use of the ultrasonic waveform (time signal) yielded the highest overall accuracy of 76% when the SVM algorithm was used. The highest overall accuracy that was obtained from frequency domain signals was 71.08% and this is from using the spectral entropy from the sampling rate of 125 kHz with the SVM algorithm. For comparison, another analysis with feature extraction was done when using the default sampling rate of 10 MHz and it still yielded an overall accuracy of 71.08%. For practicality during field inspections or testing, choosing the smallest sampling rate was appropriate since the accuracy was still the same. From these observations, the use of spectral entropy and instantaneous frequency did not improve the accuracy of machine learning classification. Furthermore, conversion of the time domain signal to the frequency domain to extract other features will add another step that may affect the reliability of the final data set since the number of features (or data points) will be reduced. In addition, the computational cost for preprocessing might be affected. In this study, a small dataset was used and the added time for preprocessing was not noticeable, but for larger datasets, this might be significant. 

#### 3.3.2. Performance Evaluation

In this section, the accuracy of the utilized classification algorithms and the different input data was analyzed in identifying the differences between concretes fired at 100 °C, 200 °C, and 300 °C—temperatures representing early fire damage in concrete specimens. The analysis for performance evaluation in this section was based on machine learning of the preprocessed ultrasonic pulse wave signals with a length of 5 ms and sampling rate of 125 kHz, as determined in the previous section. 

**Figure 14:**
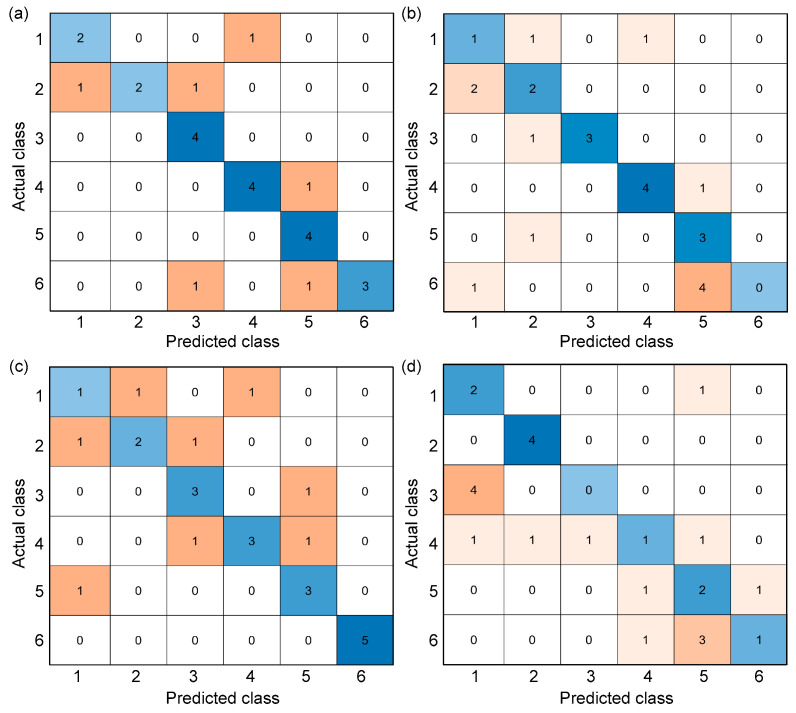
Confusion matrix of different algorithms used in machine learning for classification of thermal damage: (**a**) support vector machine, (**b**) k-nearest neighbor, (**c**) Gaussian Naïve Bayes, and (**d**) decision tree.

Figure 14 shows the confusion matrix from using different machine learning classification algorithms. Table 4 shows the accuracy and *F*1-score from the machine learning classification algorithms using the UPV time series. *F*1-score and accuracy are the most common metrics that can be used for comparison of the algorithms in classifying each target temperature [77,78,79]. SVM and KNN can identify the early damage, at least with the exposure to 200 °C at an *F*1-score of 80% and 85.7%, respectively, even when using the lowest sampling rate of 125 kHz. GNB can identify the damage at 600 °C with 80% accuracy but does not result in satisfactory accuracy levels for the damage classification at lower temperatures. From the same table, the accuracy of machine learning in detecting the damage from early exposure to fire is also relatively high for SVM and KNN. Overall, machine learning algorithms show potential in detecting early thermal damage with accuracy ranging from 68% to 92%. From Figure 11, it can be observed that SVM and KNN are both consistent in classifying the ultrasonic wave signals with the appropriate target temperature, regardless of the time window and sampling rate. GNB seemed the most inconsistent when the time window of the signal was decreased while DT showed an inconsistent accuracy when the sampling rate was decreased. From these observations, SVM is the most promising machine learning method that can be used for classifying ultrasonic waves for thermal damage.

#### 3.3.3. Comparison of Prediction Models 

**Figure 15:**
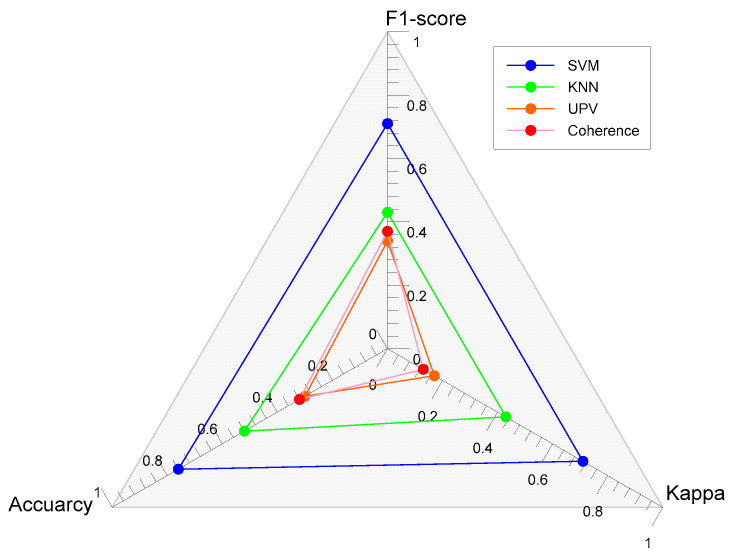
Comparison of the evaluation metrics of each method used for classifying thermal damage of concrete.

Comparing the three methods, an additional metric, Cohen’s Kappa or Kappa, was added to the overall accuracy and *F*1-score. Kappa compares the observed accuracy with an expected accuracy (random chance). In addition to evaluating a single classifier, the Kappa statistic is also used to compare classifiers. It also considers agreement with a random classifier, which means that if there is an already established thermal damage classifier or when another random classifier would be used, Kappa calculates the “agreement” between the machine learning classifier and a random classifier. Kappa also considers the imbalance in class distribution. Detailed discussion about Kappa can be read in the study of Cohen [80]. Figure 15 shows the evaluation metrics of using P-wave velocity, coherence, and two machine learning algorithms, SVM and KNN. For the use of machine learning algorithms, the following input data were used—input data of time series with a signal length of 5 ms and sampling rate of 125 kHz. Based on this comparison, it can be seen that using six classes, the best method to be used for classifying thermal damage is by utilizing machine learning with an accuracy of 76.0% and 52.0% from SVM and KNN, respectively. As discussed in Section 3.2 and Section 3.3, conventional methods of analyzing data from ultrasonic wave signals were conducted. Their results were converted to classification analysis for comparison with machine learning thermal damage classification. The use of these conventional methods from P-wave velocity and coherence yielded accuracies of 30.26% and 32.31%, respectively. It is admitted that there is still room for improvement in the performance of machine learning. However, the comparison analysis clearly demonstrates the potential of using machine learning of ultrasonic pulse waves to evaluate the early fire damage of concrete with far improved accuracy compared with conventional ultrasonic wave analysis methods.

It should be noted that there are many factors that can affect the results of this study, especially the intrinsic properties of concrete such as density, porosity, and microcracks that might develop when the concretes were exposed to high temperatures. External factors should also be considered such as humidity and temperature at the time of measurement, and coupling conditions. The study took precautions in dealing with external factors, such as the calculation of delay time to deal with the coupling condition and ensuring a consistent laboratory environment for constant humidity and temperature. As for the effects of intrinsic properties, it will be best to investigate these factors in a future study.

## 4. Conclusions

This study investigated the effect of thermal damage on the ultrasonic wave velocities of concrete. The focus was specifically on early damage detection. Conventional analysis for the UPV was done considering the linear and nonlinear parameters. Moreover, the use of machine learning, with deep learning as a supplement in improving machine learning analysis, was proposed and explored. The following conclusions were observed:

1. In general, early thermal damage (20~300 °C) of concrete, cannot be assessed accurately by the wave velocity values as they fluctuate within this range of temperatures. The behavior of the mixes differs as all mixes increased their P-wave values by 0.1% to 10.44% after exposure to 100 °C and dropped continuously until 600 °C by 48.46% to 65.80%.
2. Coherence was used as the nonlinear UPV parameter. Significant changes were observed in the concrete after exposure to 100 °C. However, between exposures from 200 °C to 600 °C, the values fluctuate in the range of 0.110 to 0.223 and reliable observations cannot be concluded for these thermally-damaged specimens.
3. Machine learning shows potential in classifying thermal damage in concrete with significantly improved performance, with an accuracy of 76.0% than those of the conventional methods using P-wave velocity and coherence, with accuracies of 30.23% and 32.31%.
4. The optimal performance of the classification was obtained using a support vector machine (SVM) compared to the other three algorithms in this study (K-nearest Neighbor, Gaussian Naïve Bayes, and Decision Tree). The optimum input type of machine learning using SVM was determined to be a time series signal with a signal length of 5 ms and a sampling rate of 125 kHz.
5. This study focused only on the effects of elevated temperature on the ultrasonic wave properties of concrete cylinders in the laboratory. To draw more general conclusions, more studies considering the effects of concrete intrinsic properties (microcracks and/or porosity) and reinforcing steel in concrete (diameter and spacing of reinforcing steel and clear cover), are still needed to further investigate the practicality of machine learning classification in various structural elements under more realistic fire scenarios.

## Figures and Tables

**Table 1:** Summary of some NDT methods for evaluation of heat-induced concrete damage from the literature.

Methodology Principle	NDT Method	Parameters and Procedures	References
Optical	Visual Inspection	observation of color change, cracking and spalling of the concrete surface	[6]
	Colorimetry	specifically used to detect color changes	[8]
Stress-waves	UPV Method	assess uniformity and quality of concrete through transmission method	[9,10]
	UPE Method	pulse-echo method that is based on the idea that amplitude of stress waves introduced into concrete are altered by the existence of cracks	[11]
	IE Method	sonic-echo or seismic-echo method where a stress pulse is introduced into an object on the available surface by a transmitter	[12,13]
Electromagnetic Wave	GPR	brief bursts of electromagnetic radiation penetrate the examined material (within a certain broad frequency band)	[14]
Sclerometric methods	Rebound Hammer	approach is based on impact loading and the propagation of stress waves	[15,16]
	Windsor Probe Test	Penetration resistance test where a hardened steel probe is driven into the concrete	[17,18]

**Table 2:** Properties of the concrete cylinders used in this study.

	Mixture Proportion (kg/m^3^)								
	W	C	S	G	SCMs		CA	W/B (%)	S_V_/A_V_
					FA	SC	AE		
MIX 1	168	219	908	931	31	62	2.18	53.85	0.497
MIX 2	170	110	858	923	37	220	2.57	46.32	0.485
MIX 3	163	230	859	887	46	184	4.60	35.43	0.495

Note W: water, B: binder, S_V_: volume of sand, A_V_: volume of aggregates, C: Portland cement type I, S: sand, G: gravel, SCMs: Supplementary cementitious materials, FA: fly ash type II, SC: Blast furnace slag cement type II, CA: Chemical Admixtures, AE: high-performance air-entraining agent.

**Table 3:** List of the machine learning algorithms used in this study.

Classification Method	Principle	Advantage	Limitations
Support Vector Machine (SVM)	▪ Classifies data by determining the optimum hyperplane for separating data points from different classes.	▪ Effective in high-dimensional spaces ▪ May be used effectively with unstructured and semi-structured data	▪ Large datasets require a long training period ▪ Resulting model is difficult to comprehend and interpret
K-nearest Neighbor (KNN)	▪ New data point is classified based on its similarity to a set of nearby data points	▪ Simple and comprehensible ▪ Resistance to noisy data among them. Provides a high level of performance	▪ In low dimensions, prediction accuracy is good, but not in high dimensions ▪ Use a lot of memory and are difficult to understand
Gaussian Naive Bayes (GNB)	▪ Given the class, Bayes theorem is used to assume that predictors are conditionally independent	▪ Simple to understand and apply to multiclass categorization	▪ Flexibility is limited ▪ To manage model flexibility, you can’t modify any parameters
Decision Tree (DT)	▪ A branching flowchart that represents several paths for possible options and results	▪ Ease of use and interpretability	▪ Predictive accuracy is low

**Table 4:** Accuracy and *F*1-score of different ML algorithms in classifying the UPV signals using sampling rate equal to 125 kHz and time window equal to 5 ms.

Classes	Accuracy				*F*1-Score			
	SVM	KNN	GNB	DT	SVM	KNN	GNB	DT
T20	0.920	0.800	0.840	0.680	0.667	0.286	0.333	0.333
T100	0.920	0.800	0.840	0.800	0.667	0.444	0.333	0.444
T200	0.920	0.960	0.760	0.840	0.800	0.857	0.250	0.000
T300	0.920	0.920	0.680	0.760	0.800	0.800	0.333	0.250
T400	0.920	0.760	0.840	0.720	0.800	0.500	0.500	0.222
T600	0.920	0.800	0.920	0.750	0.750	0.000	0.800	0.250

## Data Availability

Data are contained in this article. But the data presented in this study are also available upon request from the corresponding author.
